# Identification of risk factors for involuntary psychiatric hospitalization: using environmental socioeconomic data and methods of machine learning to improve prediction

**DOI:** 10.1186/s12888-020-02803-w

**Published:** 2020-08-08

**Authors:** O. Karasch, M. Schmitz-Buhl, R. Mennicken, J. Zielasek, E. Gouzoulis-Mayfrank

**Affiliations:** 1 LVR-Institute for Healthcare Research, Wilhelm-Griesinger-Strasse 23, 51109 Cologne (Köln), Germany; 2 LVR Clinics Cologne, Cologne, Germany; 3 LVR Clinical Group Department, Cologne, and FOM University of Applied Sciences, Essen, Germany

**Keywords:** Mental health act, Involuntary admission, Machine learning, Decision-tree analysis, Environmental socioeconomic data, Urbanization, Area-deprivation

## Abstract

**Background:**

The purpose of this study was to identify factors associated with a high risk of involuntary psychiatric in-patient hospitalization both on the individual level and on the level of mental health services and the socioeconomic environment that patients live in.

**Methods:**

The present study expands on a previous analysis of the health records of 5764 cases admitted as in-patients in the four psychiatric hospitals of the Metropolitan City of Cologne, Germany, in the year 2011 (1773 cases treated under the Mental Health Act and 3991 cases treated voluntarily). Our previous analysis had included medical, sociodemographic and socioeconomic data of every case and used a machine learning-based prediction model employing chi-squared automatic interaction detection (CHAID). Our current analysis attempts to improve the previous one through (1) optimizing the machine learning procedures (use of a different type of decision-tree prediction model (Classification and Regression Trees (CART) and application of hyperparameter tuning (HT)), and (2) the addition of patients’ environmental socioeconomic data (ESED) to the data set.

**Results:**

Compared to our previous analysis, model fit was improved. Main diagnoses of an organic mental or a psychotic disorder (ICD-10 groups F0 and F2), suicidal behavior upon admission, admission outside of regular service hours and absence of outpatient treatment prior to admission were confirmed as powerful predictors of detention. Particularly high risks were shown for (1) patients with an organic mental disorder, specifically if they were retired, admitted outside of regular service hours and lived in assisted housing, (2) patients with suicidal tendencies upon admission who did not suffer from an affective disorder, specifically if it was unclear whether there had been previous suicide attempts, or if the affected person lived in areas with high unemployment rates, and (3) patients with psychosis, specifically those who lived in densely built areas with a large proportion of small or one-person households.

**Conclusions:**

Certain psychiatric diagnoses and suicidal tendencies are major risk factors for involuntary psychiatric hospitalization. In addition, service-related and environmental socioeconomic factors contribute to the risk for detention. Identifying modifiable risk factors and particularly vulnerable risk groups should help to develop suitable preventive measures.

## Background

Involuntary hospitalization and other coercive measures are highly critical aspects of mental healthcare. They are used to handle acute situations of danger to the patients themselves or to others. However, coercive measures themselves can cause severe harm to patients and staff [1]. There is broad societal, ethical and medical consensus that the use of all kinds of coercion should be restricted as far as possible in mental healthcare [2–4].

A recent study reported a high variation in involuntary hospitalization rates across 22 European countries, Australia and New Zealand [5]. Countries with higher health care spending per capita, lower absolute poverty and a larger proportion of foreign-born individuals in the population appeared to have higher detention rates. Germany had roughly the third highest rate of involuntary hospitalization among the countries included in this study [5].

In order to target preventive interventions against involuntary admission and other coercive measures, it is important to identify modifiable risk factors, which exist on different levels and encompass patient-related clinical and sociodemographic as well as socioeconomic factors, quality of mental health services and emergency services, factors related to the social environment, and laws and the way how municipal courts and police services are organized.

On the individual level, there has been consistent evidence for people with schizophrenia or other psychotic disorders [6–8] and people with previous experience of detention [9, 10] to be at high risk for involuntary admission. There is some indication that risk may be also high for people with bipolar disorder, dementia and other organic mental disorders [11, 12], although this evidence has been less consistent. Low motivation for treatment, marked severity of symptoms, low insight into the disorder and being a danger to others were shown as common risk factors for detention among the different diagnostic groups [6, 13–16]. In terms of sociodemographic factors, male gender and migratory background have been most commonly associated with involuntary admission [6–8, 12, 17]; however, the evidence for the impact of male gender has been inconsistent across studies in different countries and continents and may depend on societal factors [13, 18]. Evidence for the impact of other sociodemographic and socioeconomic characteristics on the patient-level has been even less consistent. Altogether, there is some indication that being unemployed or homeless, receiving disability pension or social benefits, being a member of a lower social class and having a lower level of education and poor social support may be associated with higher risks for involuntary hospitalization [9, 12, 19–21].

On the level of social environment, living in urban regions with high population density and living in socially deprived areas with a high unemployment rate, small household size and high percentage of immigrants were identified as risk factors for detention [7, 9, 22, 23].

On the organizational level of mental health services, longer waiting times for regular services and lower levels of service integration were shown to increase the risk of being hospitalized involuntarily [22, 24]. Furthermore, being admitted outside of regular service hours, i.e. at night or during the weekend, was associated with compulsory admission [11, 20, 25]. Finally, on the level of legal frameworks and regulations, the mandatory involvement of a legal advisor in the procedure of detention was shown to be associated with fewer involuntary admissions in EU countries [6].

According to a recent meta-analysis, the two most important predictors for involuntary admission were a history of previous detention and a diagnosis of a psychotic disorder [10]. Other risk factors were receiving welfare benefits, being diagnosed with bipolar disorder, single marital status, unemployment and male gender (in order of effect size). Furthermore, a higher degree of socioeconomic deprivation of the living area was found to be associated with higher rates of involuntary treatment [10].

Most studies used a retrospective design and analyzed preexisting, routinely collected data from medical case and/or administrative files of one or more hospitals. Few studies analyzed data from public sources, e.g. Mental Health Act registers, or national health reports. Some studies used prospective designs analysing data from consecutively admitted cases [9, 13–16, 20, 26–28] and they included non-routine data such as ratings on symptom severity or insight and patient self-reports on perceived social support and other relevant aspects. Hence, studies using different data sources and study designs focused on different possible risk factors. To our knowledge, only one recent study of our group used machine learning (ML) procedures in order to explore the hierarchy and possible interactions between different risk factors for involuntary hospitalization [17].

In our previous retrospective study, we analyzed the health records of all persons treated as in-patients under the Mental Health Act in the four psychiatric hospitals of the metropolitan City of Cologne in Germany in the year 2011. We compared these records with the records of voluntary cases from the same hospitals and the same time period [17]. We extracted medical, sociodemographic and socioeconomic data from the records of 5764 cases and constructed a prediction model employing a decision tree method (chi-squared automatic interaction detection (CHAID)). The patient’s main diagnosis upon hospital admission was found to be the strongest predictor of involuntary hospitalization, indicating high risks for people with dementia or other organic mental disorders (ICD10: F0), schizophrenia and other psychotic disorders (ICD10: F2), and mental retardation (ICD10: F7). Other predictors were lack of outpatient treatment prior to admission, previous suicide attempts, suicidal behavior upon admission, admission outside of regular service hours, and the hospital that patients were admitted to. In addition, migratory background, marital status and professional education were also identified as risk factors for detention. The highest risks of involuntary hospitalization were found for patients with a diagnosis of organic mental disorders (ICD 10: F0) who were married or widowed, and for patients with a non-organic psychotic disorder (ICD10: F2) or mental retardation (ICD10: F7) who had a migratory background. There was some impact of the individual sociodemographic and socioeconomic factors on the risk for involuntary admission, but this impact was lower compared to the impact of the psychiatric diagnosis. The impact of environmental socioeconomic factors was not assessed, as the data set did not include any characterization of the patients´ living areas [17].

The goal of the present study is to improve the predictive decision tree model for involuntary psychiatric in-patient treatment by optimizing ML techniques and by broadening the data set to not only include factors on the individual level, but also environmental socioeconomic data (ESED) as factors that may contribute to the rate of involuntary psychiatric hospitalization. An improved predictive model should lead to more robust findings, which may be more valid. The insight derived from this analysis may help to formulate a more comprehensive risk model for involuntary psychiatric hospitalization and to design better-targeted preventive measures to reduce the rate of involuntary admissions to psychiatric hospitals.

## Methods

### Setting

Cologne is the fourth largest city in Germany with a population of about one million inhabitants. In-patient psychiatric care is provided by four hospitals and it is organized on a sectoral basis. A single Municipal Court is the deciding authority for all involuntary admissions, detentions and other coercive measures, which are carried out according to the Mental Health Act of the federal state of North Rhine Westphalia (PsychKG NRW). The PsychKG NRW applies to individuals who are mentally ill and present an immediate, severe threat to themselves or others (for details see [17]).

### Data sources

The present study combines data from two different sources.

First, we used the data of the previous retrospective study [17] which analyzed health records of 5764 cases treated in the four psychiatric hospitals in Cologne in the year 2011. Individual patients may have presented as several cases within the study period, therefore we refer to “cases” and not “patients”. The study included data of all 1773 cases under the PsychKG NRW (Mental Health Act) and of 3991 voluntary cases (random sample out of 8398 voluntary cases). Medical, sociodemographic and socioeconomic data of every case were extracted from the hospital records (main diagnosis of a mental disorder and concomitant psychiatric diagnoses according to ICD-10 [29]), suicidal behavior, previous suicide attempts, previous psychiatric treatment, guardianship, time of admission, age, gender, education, vocational and income status, living area, living situation, marital status, migratory background, etc). For a full list of the data collected and further details, see [17].

For the present study, we added ESED for the living area of each case to this data set. The ESED were obtained from RWI-GEO-GRID [30] which covers small scale information on various aspects of household structure, economic strength, house types, demography and mobility [31]. We selected eight variables that reflect economic strength, degree of urbanization and familial integration. We calculated rates per 100 inhabitants for unemployment, employment, commercial enterprises, buildings, households, residential buildings and children. In addition, we calculated the average purchasing power (in Euro) per postal code. This data was added to the original data set using the postal code of each case.

### Study design

Group differences in ESED between the voluntarily and involuntarily admitted cases were analyzed by independent Student’s t-tests.

In order to determine the best predictive model according to parsimony and fit, we first analyzed the original data set [17] and compared the CHAID algorithm used (Chi-square automatic interaction detection) with other ML algorithms such as Classification and Regression Trees (CART) and optimized hyperparameter tuning (HT). Thereafter, we analyzed a data set enriched with the added ESED using the method which was previously shown to produce the best results (CART with HT, see below). Preprocessing was performed for the training and testing datasets separately to avoid data leakage. Finally, we present the best model based on the enriched dataset in detail.

### Methods of analysis

Decision trees continuously split the dataset into groups based on the best predictor per split. The definition of ‘the best predictor’ is determined in different ways depending on the specific learning algorithm. Specifically, CHAID chooses the predictors according to the Chi-square statistic, whereas CART identifies the predictors that create the most homogenous groups as a result of the split. CART always creates binary splits, whereas CHAID may split data into as many groups as there are categories within the variable that is used to split [32, 33].

We chose four points of the previous CHAID analysis as potential areas of optimizing the analysis. Thus, we ended up comparing the original CHAID analysis [17] to four new models (refer to Table 1 for an overview of the models we analyzed):

**Table 1:** Overview of the machine learning models employed in our study. The first column refers to the model name, the second column to how class imbalance was dealt with, the third column to the ML algorithm used, the fourth column to whether hyperparameter tuning (HT) was performed, and the fifth column to whether environmental socioeconomic data (ESED) were included in the analysis

Model Name	Class Imbalance	Algorithm	Hyperparameter Tuning (HT)	ESED
Model 1	Weighting	CHAID	–	–
Model 2	Imputation	CHAID	–	–
Model 3	Imputation	CART	–	–
Model 4	Imputation	CART	✓	–
Model 5	Imputation	CART	✓	✓

#### Model 1. Chi-squared automatic interaction detection (CHAID) with weighting (CHAID analysis by [17])

When using machine learning algorithms to predict a dependent variable, in our case (in) voluntary hospitalization, the number of observations per category in the dependent variable need to be equal among categories. In our case, this means that the algorithm required equal numbers of involuntary and voluntary admissions in the dependent variable; otherwise, it would have been more efficient at predicting the majority class, i.e. the larger class. There are various methods to deal with imbalances in the number of objects in different categories of the dependent variable. The previous CHAID analysis of this dataset used class weights as a means of balancing the dataset [17].

#### Model 2. Chi-squared automatic interaction detection (CHAID) with imputation

For our current analysis, we used random oversampling in order to balance the cases in our dependent variable. Random oversampling generates additional data points for the minority class in the dependent variable at random until the balance between the classes is 1:1. For the model calculation of the CHAID, the imputation was performed for the entire training dataset. Notably, the exhaustive CHAID that was calculated for this analysis used the same parameters as the one calculated previously [17], i.e. a maximum depth of three, a minimum number of 100 cases to let a group be created, and a minimum number of 150 cases to let an existing group be split.

#### Model 3. Classification and regression trees (CART) with default parameters

We created a model using default parameters in order to establish a benchmark for the subsequent model 4. For the creation of this CART model as well as models 4 and 5, random oversampling was performed for each fold in the cross-validation, if applicable.

#### Model 4. Classification and regression trees (CART) with hyperparameter tuning (HT)

HT is a method for model optimization. It exhaustively applies combinations of given parameters in order to find the model which produces the best fit. In other words, HT is an exhaustive grid search that focused on four different parameters (Table 2). Specifically, this means that, for a particular model, a number of model permutations equal to every possible combination of variables specified in HT were calculated, i.e. 6*5^3^ = 750 permutations. Maximum depth refers to the number of splits that can be performed per branch within a decision tree. Minimum number of cases per group and split refer to the number of cases for a terminal node (group) and the node preceding the terminal one (split), respectively. Minimum impurity decrease refers to the amount of gini impurity that needs to be reduced in order for a split to occur. Gini impurity denotes the chance of classifying a randomly chosen case incorrectly. For instance, when a node is comprised of 10 involuntarily and 10 voluntarily treated cases, then the chance to classify an observation incorrectly is 50%. In this case, the gini is 0.5. The lower the gini, the purer the node and the better the classification. Ideally, a decision tree would produce only end-nodes with a gini of 0.

**Table 2:** Overview of all parameters used in hyperparameter tuning (HT) with their respective values

Parameter	Values
Maximum depth of the tree	3, 4, 5, 6, 7, 8
Minimum number of cases per groups	25, 50, 75, 100, 125
Minimum number of cases per split	50, 100, 150, 200, 250
Minimum impurity decrease	.0001, .001, .01, .1, 0

#### Model 5. Classification and regression trees (CART) with HT and environmental socioeconomic data (ESED)

To assess whether the inclusion of ESED results in an improvement of the existing models, ESED were added to the data set. Thereafter, the enriched data set was analyzed with the method previously shown to produce the best results (model 4).

### Software packages and data handling

We used IBM® SPSS Statistics® (Version 24) for the CHAID analysis and the open-source machine learning library Scikit-learn (Version 0.21.1) in Python (Version 3.7.1) for the CART analysis. Prior to any algorithm calculation, we split the sample into a training/testing and a validation set with a ratio of 70:30. This ratio was adopted from our previous analysis for comparability reasons [17]. The validation set was used only in the last step to validate the models. To evaluate the steps that require validation in order to create the model (i.e. HT), K-fold cross-validation (k = 10) on the training/testing dataset was used. The chosen evaluation metric was the area under the receiver operating characteristic curve (AUROC).

## Results

### Environmental socioeconomic data (ESED): group differences between voluntary and involuntary hospitalization

Findings are summarized in Table 3. Patients who were hospitalized involuntarily under the PsychKG NRW (Mental Health Act) lived in areas with significantly more buildings, more residential buildings, and a higher purchasing power per 100 inhabitants. Patients who were admitted voluntarily lived in areas with both a higher unemployment rate and a higher employment rate per 100 inhabitants. However, effect sizes were small. Therefore, despite their statistical significance, the clinical relevance of these between-group differences is limited. However, this finding reflects effects at the level of the entire sample and does not preclude these variables from potentially contributing to model performance in our further analysis.

**Table 3:** Environmental socioeconomic data (ESED): Group differences between cases with voluntary and involuntary hospitalization as determined by independent Student’s t-tests. Purchasing power is given in Euro. All other variables are given in rates (number per 100 inhabitants). A negative T-value indicates a higher mean for involuntary vs. voluntary cases. Degrees of Freedom were adjusted for comparisons where there were statistical differences between the variances of the two groups. For significant findings, effect sizes (Cohen’s d) between the two groups were calculated

Variable	Mean (Standard Deviation)		T	df	p	Cohen’s d
	Voluntary hospitalization	Involuntary hospitalization				
Commercial enterprises	8.47 (5.36)	8.48 (5.29)	−0.7	5198	0.504	–
Unemployment	7.77 (3.04)	7.35 (3.09)	4.6	5198	< 0.000	0.137
Employment	69.12 (3. 03)	68.88 (2.95)	2.6	3147	0.009	0.08
Buildings	14.01 (4.98)	14.69 (5.13)	−4.5	5198	<.000	0.134
Residential buildings	13.84 (4.94)	14.51 (5.09)	−4.5	5198	<.000	0.134
Households	53 (5.97)	53.04 (5.97)	−0.2	5198	0.823	–
Children	13.11 (0.58)	13.14 (0.59)	−1.5	5198	0.133	–
Purchasing power	2,158,123 (281,880)	2,203,469 (314,744)	−4.9	2787	<.000	0.152

### Validation scores in AUROC (area under the receiver operating characteristic curve)

**Fig. 1:**
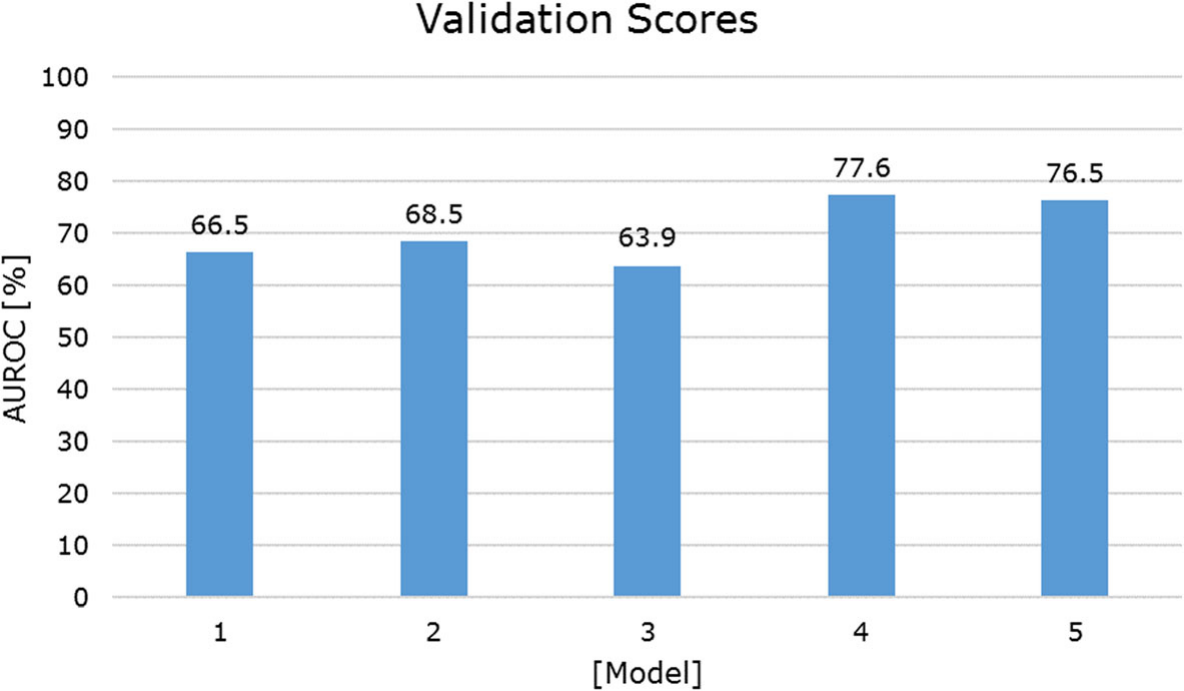
Validation scores in area under the receiver operating characteristic curve (AUROC) for model 1 [17] and for the other calculated models, i.e. the results of the present study. For an overview of the models, refer to Table 2

As indicated by validation score there was a marginal improvement of the CHAID model by using an imputation method (random oversampling) instead of weighting (AUROC of 68.5% in model 2 vs. 66.5% in model 1). Model 3 (CART with imputation, but without HT) resulted in a slightly lower validation score compared to the CHAID models 1 and 2. However, adding HT resulted in a validation score of 77.6% (model 4), which is higher than the validation score of models 1–3. Adding ESED did not lead to further improvement in terms of fit for the model. The validation score of model 5 was 1.2% lower than model 4. As this difference is marginal, both models may have similar predictive capabilities. For an overview of the validation scores in AUROC for the different models, refer to Fig. 1.

In search for the best model, we investigated possible over- and/or underfitting of models 4 and 5 by comparing testing scores with validation scores. Finally, we considered the parsimony of the models to determine the best model.

### Fit

**Fig. 2:**
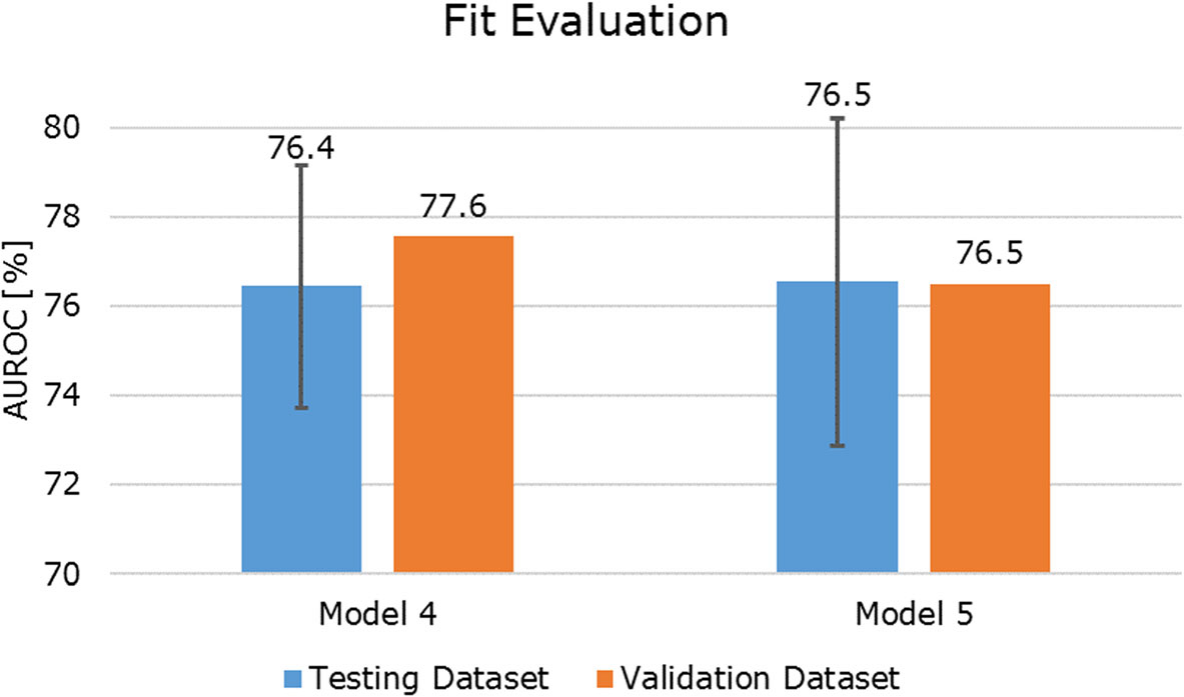
Fit evaluation for model 4 and model 5: area under the receiver operating characteristic curve (AUROC) scores [%] for the testing and validation datasets from the k-fold cross-validation. The error bars represent one standard deviation. Validation scores are point estimates (orange bars), standard deviations are therefore equal to zero. The testing dataset AUROC score refers to the testing score in the k-fold cross-validation procedure (70% of the dataset). The validation dataset AUROC score refers to the fit score of the 30% of the dataset that were split off. In case of overfitting, the validation score should be lower, and in case of underfitting it should be higher than the testing score

There was a very slight underfitting of model 4 with a validation dataset AUROC score of 77.6% and a testing dataset AUROC score of 76.4%. For model 5, the testing and validation AUROC scores were nearly identical indicating neither over- nor underfitting (76.5% AUROC score for the testing dataset and 76.5% AUROC score for the validation dataset, respectively). This analysis suggests that adding the ESED yielded a classification model that generalizes findings at least as consistently as Model 4. For an overview of the AUROC differences between the testing and validation scores of models 4 and 5, refer to Fig. 2.

### Parsimony

Table 4 gives an overview of the parameters of the models 4 and 5 which were used to estimate parsimony. Additional information on accuracy, precision and Cohen’s Kappa is summarized in Table 5.

**Table 4:** Overview of the results of hyperparameter tuning (HT) and parameter evaluation for the models 4 and 5. For the parameters used in HT, refer to Table 1. The parameter evaluation presents the two most relevant parameters for model parsimony, i.e. the total number of groups and the total number of splits

	Model 4	Model 5
**Parameter (HT)**		
Maximum depth of the tree	7	5
Minimum number of cases per groups	100	75
Minimum number of cases per split	250	200
Minimum impurity decrease	0.0001	0.0001
**Parameter (evaluation)**		
Total number of groups	54	39
Total number of splits	27	18

**Table 5:** Overview of additional parameters for the models 4 and 5. Depicted are accuracy, precision and Cohen’s Kappa with confidence intervals

Parameter [95% Confidence-Interval]	Model 4	Model 5
Accuracy	0.70 [0.68–0.72]	0.69 [0.67–0.71]
Precision	0.68 [0.66–0.70]	0.67 [0.65–0.69]
Kappa	0.37 [0.32–0.41]	0.35 [0.31–0.40]

Regarding the depth of the tree, model 5 was more parsimonious. In contrast, the required number of cases per group and per split were slightly larger for model 4, indicating a higher degree of parsimony for model 4 in that regard. The most relevant variable with regard to overall parsimony is the total number of groups and/or splits, which reflects the size and complexity of the tree in a single value: The higher the number of groups, the harder the model is to understand and the less clinically relevant the splits become. The total number of groups and/or splits was smaller for model 5, indicating a more parsimonious model.

### Decision tree of the best fitting model: model 5

**Fig. 3:**
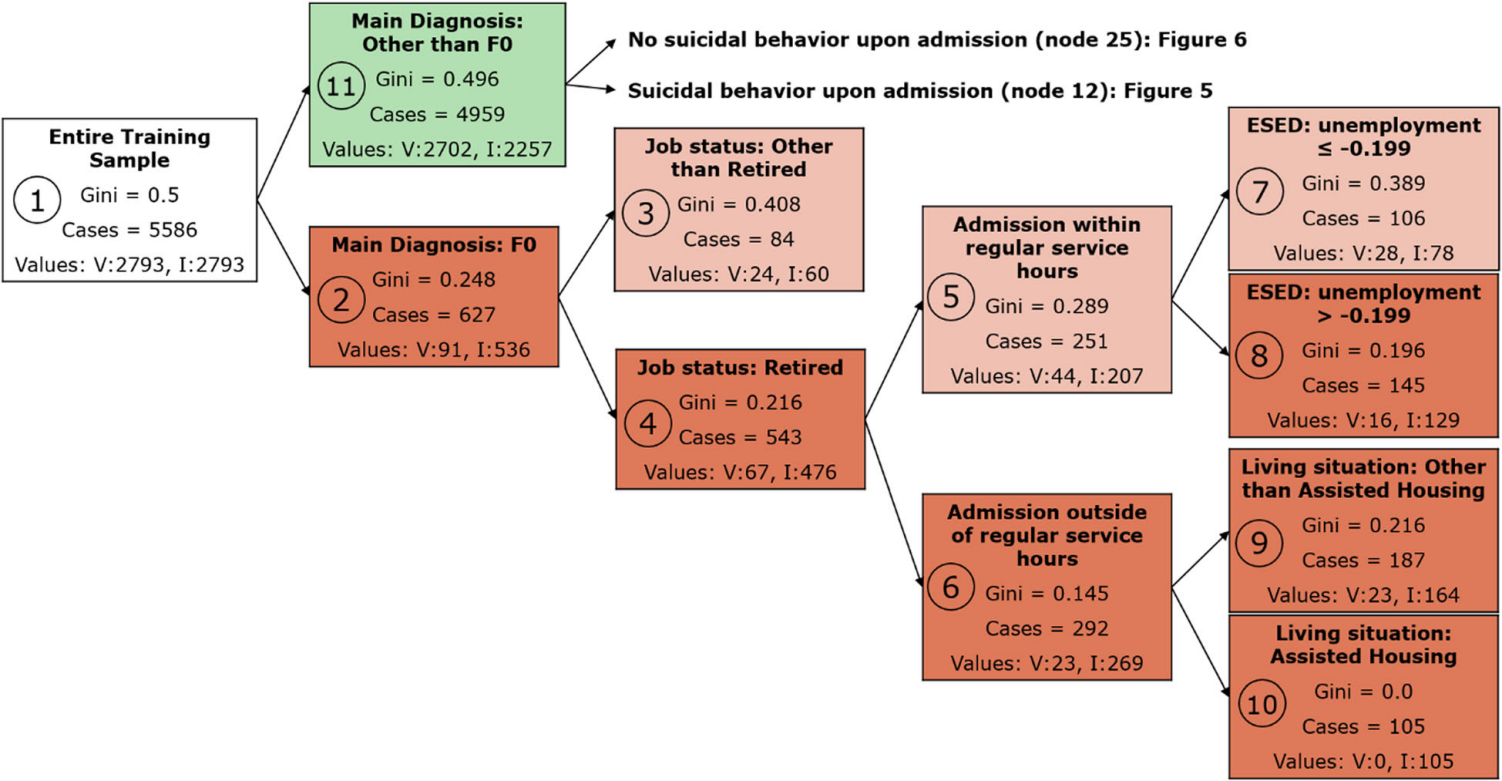
Model 5, part I: Each rectangle represents a node. The number of each node is given in the small circle within each rectangle. Node 1 includes the entire training sample. All other nodes represent subsamples that are defined by the variable given in the top line within the rectangle. Main Diagnosis: diagnosis representing the main target for diagnostics and therapy, F0: Organic mental disorders according to the ICD-10 classification. The numbers given with environmental socioeconomic data (ESED) variables (in this figure nodes 7 and 8) represent the cut-off values that were chosen by the algorithm in order to create a binary split from a continuous variable. Due to the previous standardization of the continuous variables, the unit of measurement is standard deviation (SD). With nodes 7 and 8, − 0.203 indicates that unemployment rates above or below − 0.203 SD are defined as different groups. Gini: The lower the value, the higher the purity of the node. Cases: The entire number of cases in each node. Values: The distribution of cases per node (V: number of voluntarily treated cases; I: number of involuntarily treated cases). The nodes are color coded in three different colors (red: predominantly involuntarily treated cases, green: predominantly voluntarily treated cases, white: 50/50 distribution between involuntarily and voluntarily treated cases) and two different color intensities (gini 0–0.25: strong color saturation, gini 0.25–0.5: weak color saturation). In addition, nodes are arranged in a way that, per split, the bottom node represents a larger proportion of involuntarily treated cases compared to the top node

**Fig. 4:**
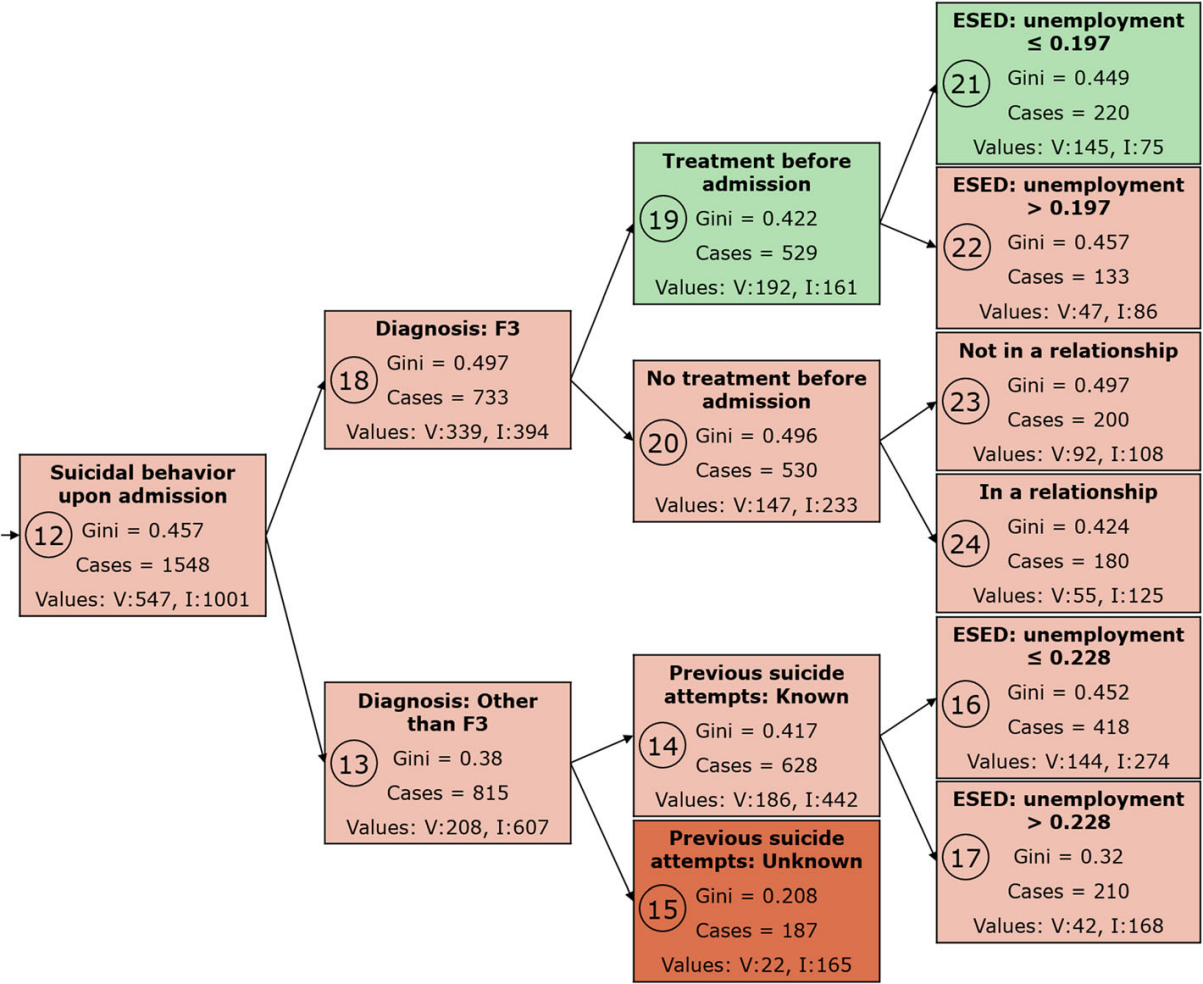
Model 5, part II. For an explanation of the figure contents, refer to the legend of Fig. 3. F3: Mood [affective] disorders according to the ICD-10 classification. ESED: environmental socioeconomic data

**Fig. 5:**
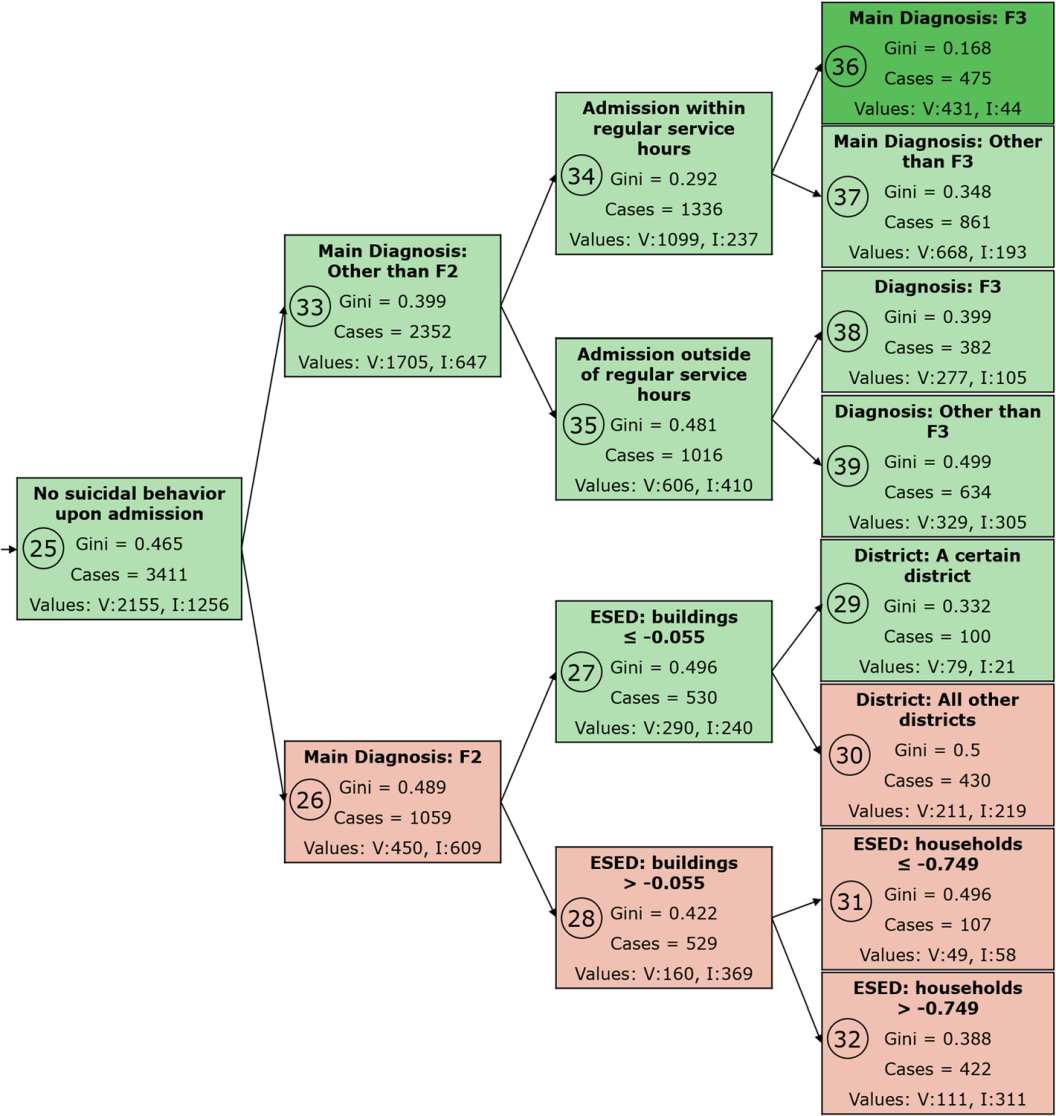
Model 5, part III. For an explanation of the figure contents, refer to the legends of Fig. 3 and Fig. 4. F2: schizophrenia or a related psychotic disorder, F3: Mood [affective] disorders according to the ICD-10 classification. ESED: environmental socioeconomic data. Note that Node 36 refers to a main diagnosis of an affective disorder (ICD-10 F3), while node 38 indicates the presence of an affective disorder, irrespective of whether it was the main or secondary diagnosis

Taken together, model 5 was the best performing model as it had a similar fit as model 4, generalized the findings from the training to the testing and validation datasets as consistently as model 4, and was more parsimonious overall. While the difference between model 5 and model 4 was small in terms of predictive efficacy, the difference in terms of parsimony indicated an overall better performance of model 5 compared to model 4. The decision tree of model 5 is presented in Figs. 3, 4 and 5.

The most important predictor of involuntary admission was whether the patient suffered from an organic mental disorder (ICD-10: F0) (Fig. 3). Cases with this main diagnosis (mostly cases with dementia and delirium) were more likely to be hospitalized involuntarily than cases with any other diagnosis (node 2). Further splits were made according to whether patients with an organic mental disorder were retired, whether they were admitted outside of regular service hours, whether they lived in an area with high unemployment, and whether they lived in assisted housing (nodes 3–10). The highest risk for involuntary hospitalization in this diagnostic group was for those who were retired, were admitted outside regular service hours and lived in assisted housing (node 10).

For those with a main diagnosis of any other mental disorder except for F0 (node 11), the most important predictor was whether suicidal behavior was present upon admission: the detention rate was higher in those with suicidal behavior upon admission (node 12) compared to those without suicidal behavior (node 25).

The best predictor of an involuntary psychiatric hospital admission for people who had a main diagnosis other than an organic mental disorder (ICD-10: F0) and who displayed suicidal behavior upon admission (node 12) was whether they suffered from an affective disorder (ICD-10: F3; nodes 13 and 18). Those who didn’t had a higher risk for involuntary hospitalization (Fig. 4).

The group of patients with an affective disorder (node 18; ICD-10 F3 diagnosis) was further divided by whether there had been out-patient treatment prior to admission (nodes 19 and 20), whether they had a partner (nodes 23 and 24), and whether the living area was one with high unemployment (nodes 21 and 22). Cases without prior out-patient treatment had a higher risk for involuntary treatment, especially when they lived in a partnership. Patients who had received out-patient treatment prior to admission had a higher risk for involuntary treatment if they lived in an area with high unemployment. The group of cases without an affective disorder (node 13) was further divided by whether the previous number of suicide attempts was known or unknown (nodes 14 and 15), and whether they lived in an area with high unemployment (nodes 16 and 17).

The most important predictor for cases without a main diagnosis of an organic mental disorder and without suicidal behavior upon admission was whether a main diagnosis of schizophrenia or a related psychotic disorder was present (ICD-10: F2) (Fig. 5). Involuntary hospitalizations were more frequent in cases with psychosis (node 26) compared to other mental disorders (except for organic mental disorders) (node 33).

Node 26 (main diagnosis F2: schizophrenia or a related psychotic disorder) was further split by two environmental socioeconomic factors (number of buildings and number of households per 100 inhabitants) and by whether or not the patient lived in a certain district of Cologne. Specifically, people with psychosis were more likely to be admitted involuntarily if they lived in a densely built area (more buildings per 100 inhabitants) with a high proportion of small households (more households per inhabitants). For cases with a main diagnosis other than schizophrenia or related psychotic disorders (node 33), the most important predictors were whether admission occurred outside of regular service hours (nodes 34 and 35), whether the main diagnosis was an affective disorder (ICD-10 F3) (nodes 36 and 37), and whether an F3 diagnosis was present, independent from whether it was the main or a secondary diagnosis (nodes 38 and 39).

## Discussion

To identify risk factors for involuntary psychiatric hospitalization, we had previously analyzed the health records of 5764 cases treated in 2011 as inpatients in the four psychiatric hospitals of the metropolitan region of the City of Cologne in Germany. We had applied CHAID as a ML algorithm and had found various risk factors on the individual level of patients and on the level of psychiatric services [17]. The current study expands on our previous work by using a different decision tree algorithm and optimization strategy to maximize model fit and by adding data on the living environment of patients to the data set. The previously created model was enhanced by the use of HT, which is a basic ML tool to determine the optimal settings for a given algorithm in order to maximize fit, and by the use of the decision tree algorithm CART. Compared to the CHAID algorithm used in our previous study, CART gives a more immediate view on which predictors are the most important ones, because it creates binary splits of the sample. In contrast, CHAID uses all available categories of a variable per split, which can be up to eight or nine categories in our data set.

As expected, the findings of our current analysis display a high degree of overlap with our previous analysis [17]. Main diagnosis, suicidal behavior upon admission, admission outside of regular service hours and absence of outpatient treatment prior to admission were identified as powerful predictors of detention in both analyses. In both analyses, people with organic mental disorders had the highest and people with psychosis had the second highest risk, while people with mood disorders had a lower than average risk for detention. Also, in both analyses people with organic mental disorders were at particularly high risk for detention when admission took place outside of regular service hours.

Other risk factors that had been previously identified on the third level of the decision tree model, such as marital status for cases suffering from an organic mental disorder, migratory background for cases suffering from schizophrenia and related psychoses, and professional education as well as the specific hospital for cases with other main diagnoses [17], were not reproduced in the current analysis. Instead, in the present analysis, people with organic mental disorders had higher risk for detention if they were retired and lived in assisted housing; and suicidal people with an affective disorder who had no outpatient treatment prior to admission had a higher risk if they lived in a relationship. In addition, the present analysis demonstrates the relevance of the living environment: The risk for detention was higher for a subgroup of cases with an organic mental disorder and for suicidal patients with several diagnoses, if they lived in areas with high unemployment. For cases with schizophrenia and other psychoses, the risk for involuntary hospitalization was higher if they lived in densely built areas, especially in combination with a higher percentage of households with few people or one-person households.

According to the present analysis, cases at particularly high risk for involuntary psychiatric hospitalization were (1) cases with an organic mental disorder diagnosis, specifically if the person was retired, admitted outside of regular service hours and lived in assisted housing, (2) cases with suicidal tendencies upon admission who did not suffer from an affective disorder, especially if it was unclear whether there had been previous suicide attempts, or otherwise if the person lived in an area with high unemployment, and (3) cases with a diagnosis of schizophrenia and related psychosis, specifically those who lived in densely built areas with a large proportion of smaller households. Overall, the present study confirms the major importance of certain psychiatric diagnoses and suicidality as risk factors for involuntary hospitalization, and, in addition, it shows that socioeconomic factors of the patients´ living environment contribute to their risk of detention.

Our findings are in line with previous research that identified unemployment as a risk factor for involuntary psychiatric hospitalization not only at the individual level, but also at an environmental, population-based level [7, 9, 10, 22, 23, 34]. Economic deprivation has also been identified as a risk factor both on the individual and the population level [10, 35]. Our findings suggest that high unemployment in the living environment may be a suitable index for overall adverse social and economic conditions associated with insufficient support of people with mental disorders. Although the nature of the association between unemployment in the environment and detention rates is not clear, living in an area with high unemployment may be considered a non-specific risk factor for detention for people with various psychiatric disorders.

A most notable finding of our current analysis is the importance of sociological factors of the living environment for cases with schizophrenia and related psychotic disorders. In our previous analysis, which had not included environmental factors, cases of schizophrenia and related disorders had been further classified according to the presence or absence of out-patient treatment, daytime of admission and migratory background [17]. In the present analysis, only the type of living environment was relevant, provided that suicidality was absent upon admission. Hence, in these cases, the living environment may be more important than individual and service-related factors. The environmental variables that were identified as risk factors for detention suggest that high degrees of urbanicity and social isolation may play a role. The fact that these variables do not appear anywhere else in the model indicate that they may be particularly critical to people with schizophrenia and related disorders.

Urbanicity has been previously shown to be associated with a higher prevalence of mental disorders and many studies have investigated this association especially in patients with schizophrenia [36–40]. Urban areas have also been associated with higher rates of compulsory in-patient treatment [35]. Our findings are in line with this evidence. Moreover, our findings extend previous evidence by showing that the association of high detention rates with urban areas refers to building density. This adds to recent findings about the importance of green and blue spaces in cities for psychological well-being [41, 42], and the association of high building volume per area with poorer mental health [43]. Finally, our finding of the impact of the average size of households in the living area as a possible index of area-level social isolation extends previous evidence on the role of being single and living alone as indices of social isolation and poor social support on an individual level [44–46] as well as findings of an association between loneliness and psychotic symptoms [47]. However, the concept of social isolation is complex in itself [48] and it is intertwined with other epidemiologic and socioeconomic factors, so that temporal and causal relationships are difficult to prove.

### Strengths and limitations

The data analyzed in this study stem from an in-depth analysis of health records of a large sample of psychiatric inpatients representative for a complete metropolitan region of Germany. As such, the present study shares a large portion of strengths with our previous analysis [17]. We analyzed the data of all Mental Health Act cases and a large sample of voluntary cases who were treated in the four psychiatric hospitals of the City of Cologne within 1 year. The region is homogenous in terms of community-based social psychiatric services provided to patients. Moreover, a single Municipal Court is responsible for all detentions. Hence, variation due to systemic reasons is minimized. However, the metropolitan region of Cologne with over 1,000,000 inhabitants is heterogeneous in relation to the socioeconomic features of the different districts and areas. The expansion of the original dataset through the addition of ESED [31] is another strength of the current study. Finally, in the current study we improved the ML analysis by using the decision tree algorithm CART and HT to optimize the prediction model.

The major limitations of our study are related to its retrospective study design. As already pointed out in our previous publication, we collected data from existing medical records and such data are always incomplete for research purposes. There was a considerable percentage of missing values for some sociodemographic variables and we had no information on potentially important variables such as symptom severity, level of psychosocial functioning, insight or perceived social support [17]. Thus, even though the data set was enriched by the ESED, these shortcomings prevent the creation of a fully comprehensive risk model for involuntary psychiatric hospitalization of people with mental disorders.

Concerning generalizability, there is some overlap between our findings and those from previous studies from Germany [11, 27] and other metropolitan regions in Europe [7, 22, 23]. Of note, a study from Switzerland which included both urban and rural regions also found that people with dementia and those with schizophrenia and related disorders were at the highest risk of involuntary psychiatric hospitalization [49]. However, numerous systemic risk factors for involuntary psychiatric hospitalization, such as quality and availability of mental health services or laws and regulations, vary grossly between countries and areas. Hence, the generalizability of our findings may be limited to Western European countries.

Finally, our data are from 2011. Since then, there have been some important changes both in the mental health system and in society which may modify detention rates and interfere with the risk factors that we aimed to identify in our analysis. Most importantly, the PsychKG NRW (Mental Health Act) was reformed. Furthermore, the refugee crisis has brought a large number of psychologically severely burdened, socially and communicatively disadvantaged people from foreign cultures into the country. Finally, the city of Cologne has been growing fast during the last 10 years, so risks associated with urbanicity may be stronger today. Although the main findings of our analysis should remain valid, analyses on newer datasets are clearly needed.

## Conclusions

Identifying modifiable risk factors is a first step in an effort to develop promising interventions which may help to reduce involuntary treatment in different risk groups of people with psychiatric disorders.

Patients with organic mental disorders had the highest likelihood of involuntary hospitalization both in our previous [17] and in the present analysis. Clearly, the diagnosis itself is not modifiable. However, admission outside of regular service hours and living in assisted housing added to the risk for detention, and these factors point to promising measures such as specific training in communication and deescalation skills for relatives and professionals who provide care at home or in residential institutions. This may help manage crises in at-home situations and avoid hospital admissions, particularly at night or during weekends [50].

Admission outside of regular service hours increased the risk of detention not only in the high-risk group of people with organic mental disorders, but also in groups with a moderate risk, specifically people with a main diagnosis other than organic mental disorder and psychosis who had no suicidal tendencies upon admission. Although the nature of the association between time of admission in a psychiatric hospital and the legal status is clearly complex, it is plausible that emergency hospital services with lower staffing levels may increase the risk of involuntary admission. Improving communication and de-escalation skills of professionals in emergency units of general and psychiatric hospitals as well as other emergency services by specific training may be crucial in reducing involuntary psychiatric in-patient admissions. In addition, concrete plans to reduce out-of-office hour consultations are needed. Specifically, the staff in living homes for the elderly may need to be better supported by psychiatric counselling. Furthermore, community-based, outreach crisis intervention services may lower the need for hospital attendance and this may lower involuntary admission rates [51, 52]. Finally, telepsychiatric counselling services may also help to improve the management of psychiatric emergencies and limit the need for psychiatric hospitalization [53]. Hence, there is a need to reinforce implementation and evaluation of telepsychiatric services for crisis intervention.

The association between socioeconomic variables of the living environment of mentally ill people and their risk for detention points to possible measures on higher-order levels. These measures may address the allocation of resources for mental health services and even aspects of urban planning. Specifically, it may be reasonable to strengthen community mental health services especially in unprivileged areas and districts with high unemployment and area-deprivation. In addition, although urbanization in terms of an increasing concentration of people in small spaces cannot be turned back, consideration of the importance of green spaces by city planners may be crucial not only for the climate and the general health and psychological well-being of people [40, 54], but also for the avoidance of stress, crises and involuntary hospitalizations in vulnerable people with psychotic disorders. Finally, urban planning and communal programs should offer possibilities for people to meet and engage in social activities. In addition, opportunities for social contacts in community mental health centers and befriending programs may help people with psychosis to overcome their problems of social isolation [55, 56]. This may also lead to better outcomes and quality of life and prevent crises and coercion. Clearly, more studies are needed in order to establish these associations.

Understanding the complex processes of mental healthcare and their interaction with individual-level factors is becoming an increasingly important domain of using large data sets and employing sophisticated statistical methods in mental healthcare research. It promises to provide more exact personalized risk and outcome models in mental healthcare research [57]. ML and decision tree analysis have recently been used to improve suicide prediction models [58] and decision trees have been used to develop prediction models for workplace sickness absence due to mental disorders [59]. Our study further advances the use of these technologies in the fields of public health and prevention. In conclusion, the models developed in our study enable us to identify promising interventions with a view to build comprehensive and effective programs to reduce involuntary psychiatric hospital admission rates in people with mental disorders. There is an urgent need to develop, implement and evaluate such programs.

## Data Availability

The dataset is available from the corresponding author on reasonable request.

## Abbreviations

AUROC: Area under the receiver operating characteristic
CART: Classification and Regression Trees
CHAID: Chi-squared automatic interaction detection
HT: Hyperparameter Tuning
LVR: Landschaftsverband Rheinland (Rhineland Regional Council)
NRW: North Rhine Westphalia
PsychKG NRW: Mental Health Act of North Rhine-Westphalia
ESED: Environmental Socioeconomic data
